# Xenomake: a pipeline for processing and sorting xenograft reads from spatial transcriptomic experiments

**DOI:** 10.1093/bioinformatics/btae608

**Published:** 2024-10-14

**Authors:** Benjamin S Strope, Katherine E Pendleton, William Z Bowie, Gloria V Echeverria, Qian Zhu

**Affiliations:** Lester and Sue Smith Breast Center, Baylor College of Medicine, Houston, TX, 77030, United States; Dan L Duncan Cancer Center, Baylor College of Medicine, Houston, TX, 77030, United States; Lester and Sue Smith Breast Center, Baylor College of Medicine, Houston, TX, 77030, United States; Dan L Duncan Cancer Center, Baylor College of Medicine, Houston, TX, 77030, United States; Department of Molecular and Human Genetics, Baylor College of Medicine, Houston, TX, 77030, United States; Lester and Sue Smith Breast Center, Baylor College of Medicine, Houston, TX, 77030, United States; Dan L Duncan Cancer Center, Baylor College of Medicine, Houston, TX, 77030, United States; Lester and Sue Smith Breast Center, Baylor College of Medicine, Houston, TX, 77030, United States; Dan L Duncan Cancer Center, Baylor College of Medicine, Houston, TX, 77030, United States; Department of Molecular and Cellular Biology, Baylor College of Medicine, Houston, TX, 77030, United States; Lester and Sue Smith Breast Center, Baylor College of Medicine, Houston, TX, 77030, United States; Dan L Duncan Cancer Center, Baylor College of Medicine, Houston, TX, 77030, United States; Department of Molecular and Human Genetics, Baylor College of Medicine, Houston, TX, 77030, United States

## Abstract

**Summary:**

Xenograft models are attractive models that mimic human tumor biology and permit one to perturb the tumor microenvironment and study its drug response. Spatially resolved transcriptomics (SRT) provides a powerful way to study the organization of xenograft models, but currently there is a lack of specialized pipeline for processing xenograft reads originated from SRT experiments. Xenomake is a standalone pipeline for the automated handling of spatial xenograft reads. Xenomake handles read processing, alignment, xenograft read sorting, and connects well with downstream spatial analysis packages. We additionally show that Xenomake can correctly assign organism-specific reads, reduce sparsity of data by increasing gene counts, while maintaining biological relevance for studies.

**Availability and implementation:**

Xenomake is an open-source program that is available on Github (https://github.com/qianzhulab/Xenomake). Complete documentation can be found at the link.

## 1 Introduction

Xenograft models, including patient derived xenografts (PDXs) and cell line xenografts, are a widely used component of cancer research for understanding tumor/stroma interactions, screening drug therapeutics, and simulating human tumor biology to understand cancer progression and therapy resistance (Hidalgo *et al.* 2014, Dobrolecki *et al.* 2016, Liu *et al.* 2023). With the rising popularity of spatially resolved transcriptomic (SRT) technologies, there is a growing need for processing pipelines that can handle reads from PDX samples. Sequencing experiments from a PDX sample often contains a mixture of reads originating from both the host and graft genomes. A unique challenge is unambiguously assigning mRNA reads as belonging to host and graft transcriptomes (Woo *et al.* 2019). This problem is especially prevalent because in order to develop into a viable xenograft, the host and graft organisms must exhibit a strong degree of homology (Batzoglou *et al.* 2000), which often leads to ambiguous mapping of reads to either organism (Woo *et al.* 2019). Currently, there are no designated tools or options in standard spatial pipelines to handle reads from PDX samples.

Previously, precise methods such as Xenome (Conway *et al.* 2012) and Xengsort (Zentgraf and Rahmann 2021) have enabled a sensitive and alignment-free way to classify PDX reads as belonging to the graft and host genomes. However, these tools have so far worked on bulk samples, and they have not been adapted to work on SRT such as 10X Genomics Visium (Ståhl *et al.* 2016, Rodriques *et al.* 2019). Adaptation to single-cell and SRT PDX data would require complex workflow modifications that are often beyond the capability of an average user. Alternative strategies such as Space Ranger (10X Genomics 2023) build an integrated reference assembly containing both host and graft genomes to which PDX reads are mapped to the organism with higher alignment score. This option however remains untested and unevaluated, and is unlikely to work well where there is high degree of homology between the host and graft.

To facilitate the adoption of SRT for PDX studies, we thus have developed Xenomake, which is an end-to-end pipeline that includes read alignment and gene quantification steps for xenograft reads generated by spatial transcriptomic platforms and uses a xenograft sorting tool to apportion these reads to the host and graft genomes. Xenomake (https://github.com/qianzhulab/Xenomake) is written based on Snakemake (Köster and Rahmann 2012, Mölder *et al.* 2021) and is fully open source. We evaluate Xenomake by conducting comparisons to show the superiority of our tool. Throughout, we demonstrate the application of Xenomake on a newly generated triple-negative breast cancer (TNBC) PDX spatial transcriptomic (ST) dataset, as well as on a published medulloblastoma PDX ST dataset.

## 2 Description

**Figure 1.**
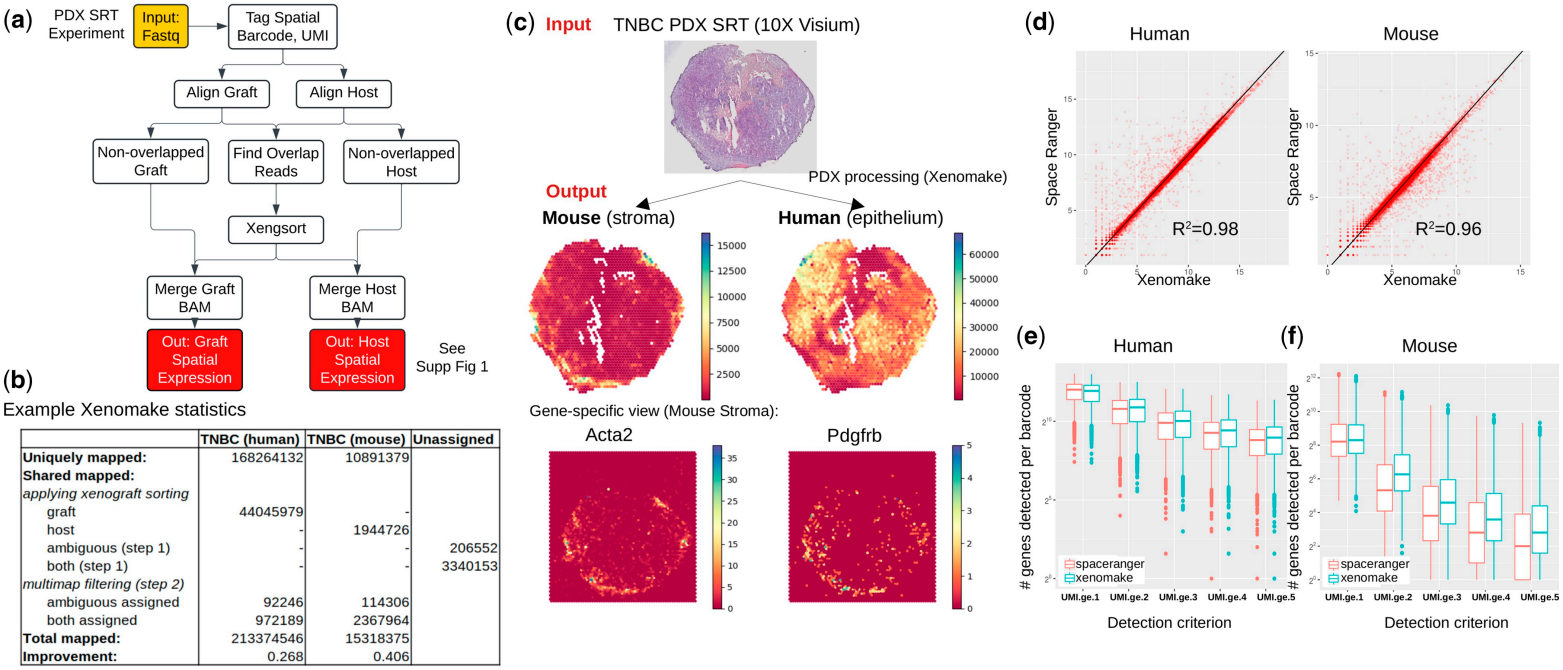
Xenomake pipeline and provided analyses. (a) Pipeline overview. Details are presented in Supplementary Fig. S1. (b) Xenomake statistics on a TNBC PDX SRT dataset. (c) Overview of input and outputs. Application on TNBC is shown. Using the outputs, users can visualize the spatial distribution of mouse (stroma) and human (epithelium) mRNA reads. (d) Comparison between the tool and Space Ranger in total UMI count (across barcodes) per gene. Each dot is a gene. Most genes are distributed below the identity line (black), indicating higher read counts assigned by Xenomake. (e, f) Comparison in total genes detected per barcode for each detection criterion: UMI ≥ 1, 2, 3, 4, and 5.

Xenomake is a xenograft reads sorting and processing pipeline adapted for SRT data. It consists of the following steps: read tagging/trimming, alignment, annotation of genomic features, xenograft read sorting, subsetting bam, filtering multi mapping reads, and gene quantifications (Fig. 1a, Supplementary Fig. S1). The input is paired-end FASTQ files. In the first step, spatial barcodes and UMI information are extracted from individual reads from FASTQ files and tagged to the reads to generate an unaligned tagged BAM file. Then, the reads are independently aligned to the host and graft genomes using STAR (Dobin *et al.* 2013). Reads that are simultaneously aligned to both genomes (called overlapping aligned reads) are next extracted and are subject to Xengsort K-mer tool (Zentgraf and Rahmann 2021) to classify them as belonging to host, graft, both, ambiguous, and neither categories. Reads in the host and graft categories are added back to the respective BAM files, while both/ambiguous undergoes further classification. The final step of the pipeline performs read multimapping handling and gene expression quantification from BAM files (Fig. 1a, Supplementary Fig. S1). The outputs are two spatial barcode-by-gene expression matrices for the host and the graft transcriptomes. For our purpose, the host refers to mouse, and the graft refers to human, as this is the common setup for PDX.

As Xengsort is performed post-alignment, no further alignment is necessary—the sorted reads and their corresponding alignments are added to the organism’s BAM file for quantification. For both/ambiguous (i.e. outputs of Xengsort), reads in these categories are often ignored and removed, but Xenomake adopts a flexible strategy to make reads in these categories usable, rather than removing them. Because the alignment location is provided for every read, our tool uses the genomic location of alignment (exonic, intronic, intergenic, or pseudogene) to determine the best location of a both/ambiguous multi-species read. For such a read, Xenomake favors the species with exonic alignment over species with intergenic, pseudogenic, and any other secondary alignments.

## 3 Results

To illustrate the capability of Xenomake, we generated an ST dataset for a previously characterized TNBC PDX model PIM001P (Echeverria *et al.* 2019, 2018) generated from a treatment naive TNBC patient who went on to exhibit therapeutic resistance and aggressive disease progression (see Supplementary Material).

Application of Xenomake returns 213 million human-aligned reads and 15 million mouse aligned reads (Fig. 1b). Of these, 168 million (78%) human reads and 10.8 million (72%) mouse reads are uniquely aligned to each organism. Xenomake conducts xenograft sorting on the 49.5 million shared aligned reads to further assign them to the source organism. This resulted in an overall improvement of 26.8% more aligned reads being assigned to human and 40.6% for mouse (Fig. 1b), compared to counting just reads uniquely mapped to each species (without leveraging xenograft sorting). We next overlayed the results to spatial positions (Fig. 1c). We were able to partition the TNBC PDX sample into mouse stroma- and human epithelial-rich regions (Fig. 1c output), with the mouse stroma surrounding the tumor. This is indicative of an invasive front that is enriched in mouse CAF populations marked by Acta2 and Pdgfra expression.

In a similar fashion, we also analyzed a recent SRT dataset focused on medulloblastoma PDX (Vo *et al.* 2023) samples in the control and palbociclib-treated setting (Supplementary Fig. S2). We similarly observed a clear division of mouse stroma-rich and human epithelial-rich regions, resembling the annotations from the paper (Vo *et al.* 2023).

### 3.1 Comparison with other tools

Space Ranger, a tool for processing 10X Genomics derived SRT samples, has been suggested to handle PDX analysis by aligning reads to the mouse-human integrated genome. In our comparison between Xenomake and Space Ranger (with the integrated genome option) on the TNBC, Xen omake mapped a total of 17 126 human genes, and 14 647 mouse genes among 2217 in-tissue barcodes. The correlation in total UMI count per gene between the two methods is very high (R2 > 0.98), but Xenomake importantly assigns more reads per gene than Space Ranger (Fig. 1d). The number of genes detected per spatial barcode is increased overall (Fig. 1e and f), and at each detection threshold of UMI count ≥ 2, 3, 4, 5, compared to Space Ranger, with the differences more prominently exhibited in mouse. Similarly in the medulloblastoma PDX, we observed an increase in the number of genes detected per barcode (Supplementary Fig. S3). Of note, the increased reads observed in Xenomake is due to the use of xenograft sorting to unambiguously assign reads to the right organism (72%–85%), and to the improved handling of ambiguous/both reads that rescue more reads (15%–28%) (Supplementary Fig. S4). Taken together, these results suggest that Xenomake reduces the sparsity of the gene expression matrix by increasing read counts on a per gene and per spot level.

Furthermore, Xenomake can more accurately attribute to the right organism those closely homologous read sequences than Space Ranger. To see this point, we focus on analyzing the most discrepant genes quantified by the two pipelines (Supplementary Fig. S5). Indeed, in the mouse compartment, the top 50 genes ranked high in Xenomake (and low in Space Ranger) encompass important transcriptional regulators and RNA processing genes such as Satb1, Bcl11a, Foxc1, Tox, Ptbp2, and Hnrnpd (Supplementary Fig. S5a). Xenomake will be beneficial for analyses where these genes are concerned. We further validated the Xenomake-high genes by checking their expression levels in a single-cell RNAseq breast cancer atlas (Supplementary Fig. S5b): 17 genes are enriched for stroma cell-type specific expression in endothelial cells and myeloid cells in the scRNAseq atlas (see Supplementary Fig. S5c red boxes). In contrast, many Space Ranger-high mouse genes are also expressed highly, but incorrectly, in cancer epithelial cells (see Supplementary Fig. S5d blue box), suggesting possible nonspecificity of some of Space Ranger output. Indeed, Xenomake generally displays a lower Shannon entropy score, meaning higher cell-type specificity, than Space Ranger (Supplementary Fig. S5e) for the stroma genes that it detected, particularly toward endothelial, myeloid, PVL cell types (Supplementary Fig. S5f). Therefore, when discrepant gene expression levels exist between the two pipelines, Xenomake’s results are more likely to find additional support from scRNAseq datasets.

### 3.2 Application: finding stroma- and epithelium-biased cell-type markers and cytokines

Xenomake generates plots to enable users to compare the mouse and human homolog expression on its gene expression matrix outputs, for any genes. For example, one may wonder if cell-type markers are stroma-biased or epithelium-biased. One can plot human and mouse homolog expression against each other (Supplementary Fig. S6). For canonical markers of stroma cell types, we expect their expression should be low in human (i.e. representing epithelium compartment), but high in mouse (i.e. stroma). Expectedly, in the TNBC PDX dataset, Pecam1, Fcgr3, Csf1r expression, which respectively mark endothelial cells, NK/neutrophils, and macrophages, are high in the mouse portion, but lowly expressed in the human counterparts (PECAM1, FCGR3A, CSF1R) (Supplementary Fig. S6a). This corroborates with the fact that Xenomake assigns correctly many stromal exclusive transcripts to the mouse genome. Conversely, if known genes that are specific to epithelium, their expression should be high in human and low in mouse. Expectedly, collagen Col1a1, an abundant protein in the extracellular matrix (ECM), is much higher than COL1A1 in human (Supplementary Fig. S6a). Using a similar idea, we also have been able to identify cytokines that are primarily expressed in human (indicating epithelial-biased expression), such as CCL28 and CCR10, and those expressed in mouse (indicating a stroma bias), such as Il33, Il10ra, Cxcl14, and Cxcl12 (Supplementary Fig. S6b). Delineation of these is useful for mining cross-compartment cytokine interactions. Overall, Xenomake can identify not only cell type markers expressed in each compartment, but also stromal and epithelial specific cytokines.

### 3.3 Application: spatial ligand–receptor interaction analysis

**Figure 2.**
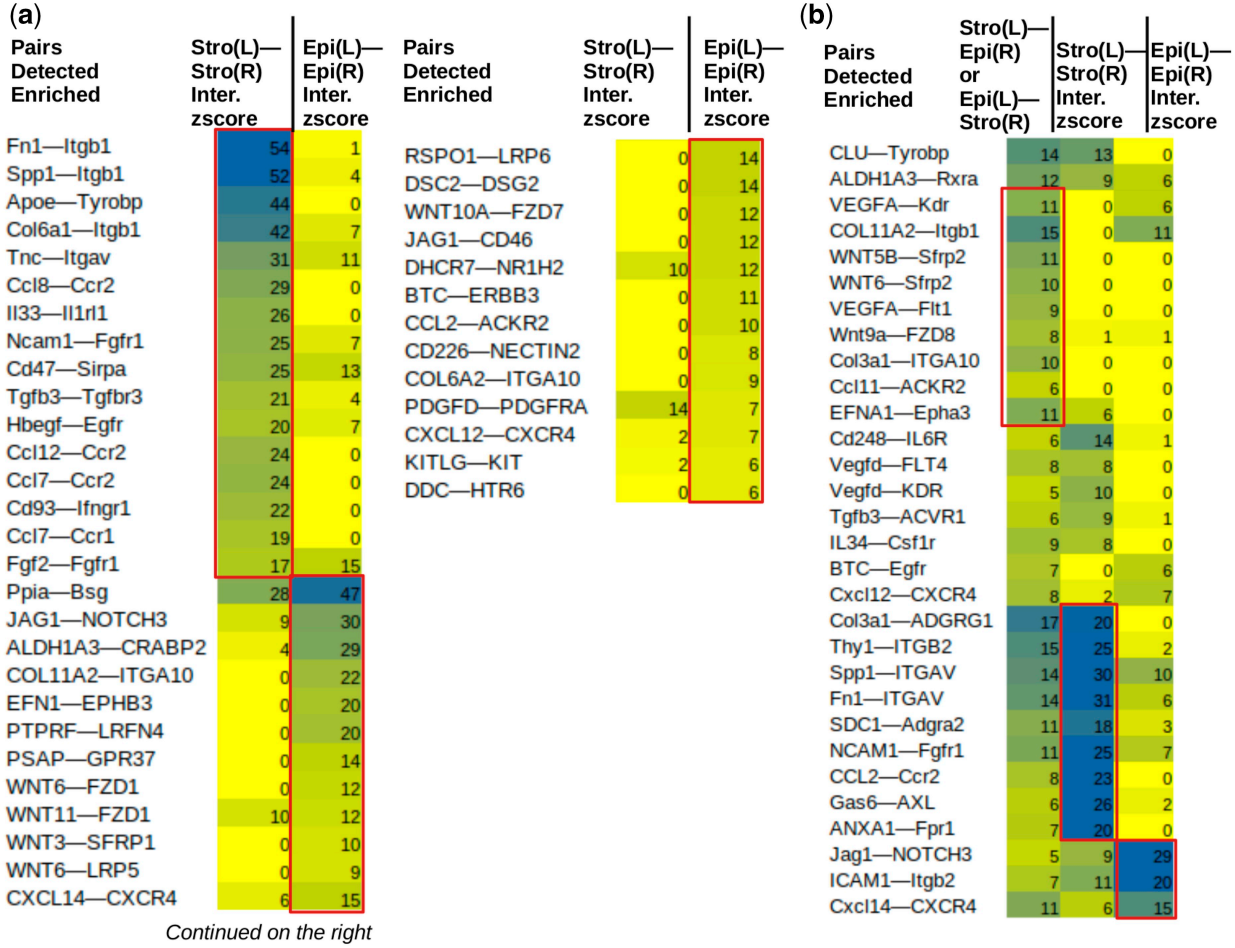
Ligand–receptor analysis. For stroma-stroma (a), epithelium-epithelium (a), and stroma-epithelium ligand receptor interactions (b). Top ligand–receptor pairs are shown. Interaction z-scores are indicated. Boxed: specific pairs. In the heading, L: ligand, R: receptor. As an example, for Fn1-Itgb1, Stro(L)-Stro(R) tests the spatial interaction between Fn1 ligand and Itgb1 receptor both in stroma compartment. Epi(L)-Epi(R) for the same pair tests interaction between FN1 ligand and ITGB1 receptor both in epithelium (genes capitalized as they are from human reads).

Using spatial information, one can further detect cell–cell communications mediated by the stroma and epithelium compartments (Fig. 2a and b). Communication is defined by the spatial co-localization of ligand and receptor gene expressions either on the same spot, or on adjacent spots connected by an edge in the spatial graph. Xenomake enumerates all possible ligand–receptor pairs, from a database such as CellPhoneDB (Efremova *et al.* 2020), to find spatially enriched within- and cross-compartment ligand–receptor interactions. These include Stroma—Stroma (SS), Epithelium—Epithelium (EE), and Stroma—Epithelium (SE) interactions (Fig. 2a and b). In TNBC, fibronectin—integrins (e.g. Fn1—Itgb1), and cytokine—cytokine (e.g. Ccl8—Ccr2) represent the predominant form of SS interactions (Fig. 1g). This forms a stark contrast with EE interactions that primarily concentrate on Notch and Wnt signaling (JAG1—NOTCH3 and WNT6—FZD1) (Fig. 2a). Xenomake also found evidence of SE interactions (Fig. 2b), such as Wnt9a—FZD8, VEGFA—Flt1, and Fn1—ITGAV, suggesting that despite the species difference of compartments in PDX, the stroma and epithelium can still communicate extensively.

## 4 Discussion

Conventional analyses of bulk PDX samples have removed or ignored mouse-assigned reads. In SRT however, there is an opportunity to study the spatial interactions between the mouse stroma and human tumor cells. Properly assigning PDX reads to each respective organism becomes important for inferring stroma-epithelial interactions and isolating the contribution of mouse stroma in shaping the tumor microenvironment. Xenomake’s results will permit an accurate delineation of stroma cell types and an understanding of cytokine signaling mediated by the mouse stroma.

A key difference between Xenomake and previous Xenome/Xengsort is that Xenomake can properly recognize and handle cellular barcodes/spatial barcodes and unique molecular identifier (UMI) (Smith *et al.* 2017) information from read sequences to correctly apportion reads to organisms and spatial locations. This allows the tool to support SRT datasets. In future we plan to support more SRT technologies (Zhu *et al.* 2018, Liu *et al.* 2020, Stickels *et al.* 2021, Chen *et al.* 2022, Cisar *et al.* 2023) as these technologies become utilized for PDX studies. Because on average between 25 and 35% of aligned reads in a PDX experiment are commonly aligned between human and mouse, using a xenograft-sorting enabled pipeline to disambiguate these assignments will bring substantial improvement to downstream spatial transcriptomic analyses (Wolf *et al.* 2018, Dries *et al.* 2021, Hao *et al.* 2021, Sztanka-Toth *et al.* 2022, Domanskyi *et al.* 2024).

## Supplementary Material

btae608_Supplementary_Data

## Data Availability

Xenomake is maintained on the Github repository located at https://github.com/qianzhulab/Xenomake/. Installation of Xenomake requires a Conda environment and Snakemake. More details can be found at the repository website. Medulloblastoma PDX data can be accessed at the accession ID E-MTAB-11720 on ArrayExpress (https://www.ebi.ac.uk/biostudies/arrayexpress/studies/E-MTAB-11720). Raw data for TNBC PDX generated in this study have been deposited on Zenodo and can be accessed using the link: https://zenodo.org/record/8313189.
