# Supplementary material for: Xenomake: a pipeline for processing and sorting xenograft reads from spatial transcriptomic experiments
Source: Bioinformatics. 2024 Oct 14;40(11):btae608. doi: 10.1093/bioinformatics/btae608 (PMC11583937; doi:10.1093/bioinformatics/btae608)
Supplement: btae608_Supplementary_Data [file btae608_supplementary_data.zip › Xenomake.final.supplementary.docx]

**Xenomake Supplementary Material**

**Materials and Methods**

**PIM001P PDX Model**

PIM001P was obtained from the University of Texas MD Anderson Cancer Center where it was generated and characterized through a materials transfer agreement. PIM001P was propagated in mice as previously described (Garrido-Castro *et al.*, 2019). In brief, frozen PDX cell suspensions were thawed and washed with Dulbecco’s modified Eagle’s medium (DMEM):F12 (Cytiva HyClone, SH30023.01) supplemented with 5% FBS. Cells were stained with AOPI (Nexcelom Bioscience, CS2-0106) to count viable cells on a Cellometer K2 (Nexcelom Bioscience). 0.5 million viable tumor cells were suspended at a 1:1 ratio in Matrigel (Corning, 354234). Suspended cells were then injected into the fourth mammary fat pads of NOD/SCID mice [NOD.CB17-Prkdcscid/NcrCrl, Charles River, National Cancer Institute (NCI) Colony] aged 5 weeks old.

**Spatial Transcriptomics of PIM001P: Library Preparation and Sequencing**

The tumor was harvested 2 days after it obtained a volume of 150mm3. At time of harvest, a thin slice of fresh tumor was cut and placed in a cassette. The cassette was then filled with O.C.T. (Sakura Finetek, Catalogue 4583) and submerged in isopentane until frozen solid. Frozen tissue cassettes were stored at -80C.

Tissue sections of 10µm thickness were mounted onto the capture areas of the Visium Spatial Gene Expression slide and stained using hematoxylin and eosin. Tissue sections were permeabilized on a thermocycler for 24 minutes, as determined by the Tissue Optimization step. Poly-adenlyated mRNA is released and captured by surface-bound primers within each capture area. Reverse transcription, template switching, extension, and second strand synthesis are performed on the slide. Full-length, spatially barcoded cDNA transcripts are then denatured from the slide and amplified via PCR prior to library construction. Approximately 110 to 375 ng of amplified cDNA was carried forward into library construction. During library construction, cDNA is enzymatically fragmented to target amplicon size then undergoes end repair, A-tailing, adapter ligation, and then amplified using between 14 and 16 PCR cycles. The resulting libraries were quantitated using the Invitrogen Qubit 2.0 quantitation assay and fragment size assessed with the Agilent Bioanalyzer. A qPCR quantitation was performed on the libraries to determine the concentration of adapter ligated fragments using Applied Biosystems ViiA7 Real-Time PCR System and a KAPA Library Quant Kit (p/n KK4824). All samples were pooled equimolarly and re-quantitated by qPCR, and also re-assessed on the Bioanalyzer.

150 pM of equimolarly pooled library was loaded onto the NovaSeq 6000 S4 flowcell and sequenced at the recommended 28-10-10-50 read configuration. PhiX Control v3 adapter-ligated library (Illumina p/n FC-110-3001) was spiked-in at 2% by weight to ensure balanced diversity and to monitor clustering and sequencing performance. A minimum of 300 million read pairs per sample was sequenced. FastQ file generation was executed using 10X Genomics’ Space Ranger mkfastq software.

**Xenomake Pipeline Implementation:**

Input:

Our pipeline requires paired end FastQ files as input for our pipeline and we recommend using Space Ranger (10X Genomics, 2023) mkfastq to make this conversion from raw Illumina base calls. We utilize Picard‘s (Broad Institute) “FastqToSam” to convert FastQ files to an unaligned BAM file. This format allows preprocessing such as tagging and trimming of reads prior to alignment.

Transcript Tagging:

Unique molecular identifiers (UMIs) and cell barcode information is present in predictable locations of forward reads as part of illumina chemistry. This positional information can be utilized to extract read segments and tag both paired end reads. For example, UMIs are always found in positions 17-28 and cell barcode information is found at positions 1-16 on forward reads. Drop-seq (Macosko *et al.*, 2015) provides the function TagBamWithReadSequenceExtended to extract bases and create a new BAM tag with those bases on the genome read. For cell barcode tagging, we use the following parameters; "BASE_RANGE=1-16 TAG_NAME=CB". For UMI tagging we use "BASE_RANGE=17-28 TAG_NAME=MI”

Adapter and PolyA Trimming:

The Drop-seq (Macosko *et al.*, 2015) program provides tools to remove adapter sequences from 3’ ends of reads and polyA stretches using TrimStartingSequence and PolyATrimmer respectively. PolyATrimmer is designed to trim polyA tails from reads by searching for at least 6 contiguous bases at the 3’ end with zero mismatches and hard clips of these bases (MISMATCHES=0, NUM_BASES=6) Additionally TrimStartingSequence searches for 5 contiguous bases with zero mismatches in a provided Illumina adapter sequence (SEQUENCE=Adapter_Sequence MISMATCHES=0 NUM_BASES=5) and hard-clip the bases off the read if they occur at the 5’ end.

Mapping:

Mapping is performed via STAR (Dobin *et al.*, 2013) on tagged and trimmed unaligned bam files. We utilized default mapping and indexing parameters for genome assemblies provided by STAR and output an unsorted bam file. Since STAR doesn’t preserve cell barcode and UMI tags, these need to be merged from the unaligned bam file to the aligned bam output. A custom python script is used to merge tags to generate a tagged and aligned bam file. Subsequently, the Drop-seq (Macosko *et al.*, 2015) program TagReadWithGeneFunction is used with default parameters to tag reads that are exonic when the read overlaps the exon of a gene. This tag contains the name of the gene as reported in the provided annotation file and a tag from the following list [INTERGENIC, INTRONIC, UTR, CODING, RIBOSOMAL].

Xenograft Sorting:

The core of Xenomake is the implementation of xenograft sorting on reads that are mapped to both mouse and human organisms (overlapped reads). Xenograft Sorting is performed using Xengsort (Zentgraf and Rahmann, 2021), a faster and lightweight implementation than previous methods such as Xenome (Conway *et al.*, 2012). Xengsort is an alignment-free method that stores k-mers into a large table that enables the lookup of species of origin (host,graft, or both) of each RNA k-mer that occurs in either genome assembly. Reads are classified by iteratively searching k-mers for the appropriate species of origin. This process assigns reads into “host”, “graft”, “ambiguous”, “both”, or “neither” based upon Xengsort’s decision rule. When building the index files used in classification, Xengsort recommends storing 4.5 billion 25-mers when using humans and mouse genomes. After index generation, we implement Xengsort classify on our reads using default settings.

Reassignment of Xenograft Sorted Reads:

We can confidently reassign reads back into the human or mouse alignment files on xenograft sorted files (graft and host respectively). Our idea for handling multimapped reads is inspired by another spatial processing tool, Spacemake (Sztanka-Toth *et al.*, 2022). Briefly, we wrote a Python script that can assign the “ambiguous” and “both” output reads back into respective organisms. It compares gene function tags and STAR alignment scores (AS) to determine which organism this read should be reassigned. Briefly, a read is compared between both organism’s alignment files side by side and makes a call based upon the following strategies. First, if only one of the reads aligns to an exonic region, then it is reassigned to that organism. Second, if the AS differs between two organisms, the read with a higher AS is reassigned. If neither requirement is satisfied, the read is discarded. This method leverages information from mapping, functional tags, and alignment free sorting to recover as many reads as possible.

Within-species Multi-Mapped Read Assignment:

Handling multi-mapped reads within species works by filtering reads that are multi-mapped to different genomic positions by STAR (Dobin *et al.*, 2013). Its decision rule is identical to the reassignment of xenograft reads but does no organismal comparison. Again, it prioritizes reads that are mapped to exons, have a higher alignment score, and removes the “worse” read from the alignment. This is an optional parameter but removes the potential to count reads twice in downstream processing.

Output Files:

We offer multiple options for files used in downstream analysis. First, a gene by spot umi count matrix is generated using the Drop-seq (Macosko *et al.*, 2015) program DigitalGeneExpression. This takes the gene tags, cell barcode tags, and UMI tags to develop a count matrix in tab delimited format. We also implement a custom script that replicates the 10X genomics HDF5 architecture. Tools such as Scanpy (Wolf *et al.*, 2018), Giotto (Dries *et al.*, 2021), and other spatial pre-processing can take HDF5(The HDF Group) spatial files as input and allow easier integration for downstream analysis by users. Additionally, we also utilize Scanpy to generate H5ad objects that can be loaded directly by multiple spatial packages.

Downstream Processing:

We utilize the H5ad object and a Scanpy workflow (Wolf *et al.*, 2018) to provide basic downstream analysis of spatial samples as an optional parameter. As a part of this, we offer an estimation for in-tissue segments in-silico using KMeans clustering. We set the number of clusters a priori to two and grouped all spots into these categories. Fundamentally, spots with low expression and low number of genes expressed should group together; conversely spots with high counts and higher number of genes should cluster and represent in-tissue segments.

We further process samples by filtering spots that are designated out-of-tissue. We subsequently find highly variable genes (HVGs) using sc.pp.highly_variable_genes and use a subset of HVGs to perform principal component analysis (sc.pl.pca), UMAP projection on the top 40 PCs (sc.pl.umap) and perform Leiden clustering with default parameters (sc.pl.leiden). The output is saved as an Anndata object and can be loaded with spatial transcriptomics packages such as Scanpy and Squidpy.

**Xenomake Comparisons:**

Comparison with Space Ranger

We downloaded Space Ranger version 2.0.1 from the 10X Genomics website. Following the recommended tutorial, we built a reference for multiple species by running the “spaceranger mkref” command with the two reference genome assemblies being mm10 (version M23 Ensembl 98) and GRCh38 (version 32 Ensembl 98). We next used “spaceranger count” with default settings to align reads to the integrated reference genome and quantify gene expression. The output of Space Ranger attached “hg38_” and “mm10_” prefixes to each human and mouse gene name, allowing further division into individual species. The capture area information, i.e., A1-C1, was available from the Visium slide for TNBC PDX and on the raw data repository for the medulloblastoma PDX. Following quantification, we extracted in-tissue barcodes and all genes with nonzero read counts in these barcodes to use them for comparison purpose. For per-gene and per-barcode comparisons, we summed gene expression across in-tissue barcodes for each gene, and summed gene expression across all genes for each barcode. For the number of genes detected per barcode, we set a gene expression detection threshold criterion to 2-5 UMI counts and counted the number of genes exceeding the detection threshold. When making comparisons between methods, we counted the number of cases in the three categories: (1) Equal: Xenomake and Space Ranger gene counts are the same, (2) X>S: Xenomake returns higher gene counts than Space Ranger, and (3) S>X. Intuitively, (1) will indicate agreement between methods, while (2) relative to (3) will indicate the superiority of Xenomake in terms of increased read depth.

Comparison Using Single-Cell RNAseq

We downloaded a breast cancer single cell atlas from (Wu *et al.*, 2021). Cell type specific gene expression was visualized using a dot plot. To extract discrepant genes between Xenomake and Space Ranger, we determine the top 50 genes, in each direction, that lie furthest from the diagonal line in the correlation plot. These genes were labeled in Supplementary Figure 1. To compute cell type specificity score for a gene *g*, we utilized the Shannon Entropy Index which is defined as:

$$H_{g}=\sum-p_{g,c}{log}_{10}p_{g,c}$$

Where *Hg* is the entropy of *g*, *c* is a cell-type, *pg,c* is the probability of expression of *g* in cell-type *c*, computed as the sum of expression of *g* in all single cells in cell-type *c* divided by the sum of expression of *g* in all cells.

**Xenomake Application: Finding Stroma- and Epithelium-Biased Genes and Cytokines**

Xenomake plots homologous gene expression against each other to find stroma- and epithelium-biased genes. We extracted (mouse, human) orthologous gene pairs from BioMart function of Ensembl website. Then for each (g­_human_, g_mouse_) pair, we summed the expression of g_mouse_ over all in-tissue barcodes in the mouse spatial gene expression matrix, and did the same for the human gene expression matrix for g_human_. We plotted the human homolog expression against the mouse homolog expression. This was done for cell-type markers being the genes of interest, and for cytokines. The list of cytokines included all interleukin-related and cytokine-related genes using a regular expression tool that detects “IL”, “CCL”, “CCR”, “CXCL”, and “CXCR” in gene names. When plotting the expression of human homolog against the mouse homolog, those homologs that exhibit +/- 2 standard deviations from the diagonal line in the scatter plot were deemed to have an epithelial or stromal bias.

**Xenomake Application: Spatial Ligand-Receptor Analysis**

We extracted all the ligand-receptor gene pairs from the database CellPhoneDB (Efremova *et al.*, 2020). We borrowed the same spatial ligand-receptor analysis from Giotto (Dries *et al.*, 2021) that we co-authored in 2019, but re-implemented it in Python for the purpose of this paper. A Delauney spatial graph is first constructed from the spatial positions of all spots. Then, for each gene g’s spatial expression vector [g_s1_, g_s2_, …, g_si_] where s_i_ is a Visium spot, we adopt the following procedure to binarize the gene expression vector into 1’s and 0’s (representing expression-high and expression-low groups). This is needed because the spatial colocalization test between ligand and receptor genes requires discrete spot labels. Before proceeding, we removed from ligand-receptor analysis all genes where the number of expressed spots (i.e. greater than 0 read count) is smaller than 10. For all other genes, below is the algorithm:

Algorithm 1: Get high-expression cluster

Input: gene expression vector of g across spots: v_g_ = [g_s1_, g_s2_, …, g_si_]

1. Perform K-means on v_g_ (K=5 and num_starts=1000), producing a set of spot clusters, c_1_, c_2_, … c_5_.
2. Sort clusters by cluster expression average, from highest to lowest, producing c_h1_, c_h2_, …, c_h5_, where avg(c_h1_)>avg(c_h2_)>avg(c_h3_)…
3. Set merged cluster c_m_ to be initially c_h1_.
4. Loop:
5. If c_m_>0.10*n_tot_spots_:
6. Stop. Return c_m_.
7. c_m_=c_m_ + c_h{i+1}_
8. End Loop.
9. Return c_m_

This procedure will return a high-expression cluster that is derived from successively merging the highest clusters, such that the merged high-expression cluster has as close as possible 10% of spots, to be used for co-localization test.

Next, between two genes’ high-expression spot clusters c_m1_, c_m2_, we conduct the spatial co-localization test as follows:

Algorithm 2: Spatial co-localization

Input 1: c_m1_ and c_m2_, which are the high-expression spot clusters of g_1_ and g_2_ respectively

Input 2: Spatial graph

1. Set count = 0
2. For each spot s_1_ in c_m1_:
3. For each spot s_2_ in c_m2_:
4. If there is an edge s_1_—s_2_ in the spatial neighbor graph, count = count +1
5. Return count

The above algorithm will return the observed number of spatial adjacencies between two genes’ high-expression spots. To perform statistical testing, we randomly shuffled the spots among the in-tissue spot positions, thus destroying the mapping relationships between spots and the locations. We repeated this 1000 times, and derived a distribution of random number of spatial adjacencies expected of the two genes. We computed a z-score by z = (observed – random_mean_) / random_std_, where random_mean_ and random_std_ are the mean and standard deviation of the random number of spatial adjacencies.

The above is performed between all pairs of ligand-receptor genes in CellPhoneDB database. For within-compartment interactions (stroma-stroma and epithelium-epithelium), one species (either mouse or human)’s gene expression matrix was used for measuring ligand-receptor expression interaction. For cross-compartment (i.e. stroma-epithelium) interactions, we perform interaction test between a mouse ligand gene and a human receptor gene, or alternatively between a mouse receptor gene and a human ligand gene.

**References**

10X Genomics (2023) What is Space Ranger? -Software -Spatial Gene Expression -Official 10x Genomics Support.

Batzoglou,S. *et al.* (2000) Human and Mouse Gene Structure: Comparative Analysis and Application to Exon Prediction. *Genome Res*, **10**, 950.

Broad Institute GitHub - broadinstitute/picard: A set of command line tools (in Java) for manipulating high-throughput sequencing (HTS) data and formats such as SAM/BAM/CRAM and VCF.

Chen,A. *et al.* (2022) Spatiotemporal transcriptomic atlas of mouse organogenesis using DNA nanoball-patterned arrays. *Cell*, **185**, 1777-1792.e21.

Cisar,C. *et al.* (2023) A unified pipeline for FISH spatial transcriptomics. *Cell genomics*, **3**.

Conway,T. *et al.* (2012) Xenome--a tool for classifying reads from xenograft samples. *Bioinformatics*, **28**.

Dobin,A. *et al.* (2013) STAR: ultrafast universal RNA-seq aligner. *Bioinformatics*, **29**, 15–21.

Dobrolecki,L.E. *et al.* (2016) Patient-derived xenograft (PDX) models in basic and translational breast cancer research. *Cancer and Metastasis Reviews 2016 35:4*, **35**, 547–573.

Domanskyi,S. *et al.* (2024) Nextflow pipeline for Visium and H&amp;E data from patient-derived xenograft samples. *Cell Reports Methods*, **4**, 100759.

Dries,R. *et al.* (2021) Giotto: a toolbox for integrative analysis and visualization of spatial expression data. *Genome Biol*, **22**, 1–31.

Echeverria,G. V. *et al.* (2018) High-resolution clonal mapping of multi-organ metastasis in triple negative breast cancer. *Nature Communications 2018 9:1*, **9**, 1–17.

Echeverria,G. V. *et al.* (2019) Resistance to neoadjuvant chemotherapy in triple-negative breast cancer mediated by a reversible drug-tolerant state. *Sci Transl Med*, **11**, 936.

Efremova,M. *et al.* (2020) CellPhoneDB: inferring cell–cell communication from combined expression of multi-subunit ligand–receptor complexes. *Nature Protocols 2020 15:4*, **15**, 1484–1506.

Garrido-Castro,A.C. *et al.* (2019) Insights into molecular classifications of triple-negative breast cancer: Improving patient selection for treatment. *Cancer Discov*, **9**, 176–198.

Hao,Y. *et al.* (2021) Integrated analysis of multimodal single-cell data. *Cell*, **184**, 3573-3587.e29.

Hidalgo,M. *et al.* (2014) Patient Derived Xenograft Models: An Emerging Platform for Translational Cancer Research. *Cancer Discov*, **4**, 998.

Köster,J. and Rahmann,S. (2012) Snakemake—a scalable bioinformatics workflow engine. *Bioinformatics*, **28**, 2520–2522.

Liu,Y. *et al.* (2020) High-Spatial-Resolution Multi-Omics Sequencing via Deterministic Barcoding in Tissue. *Cell*, **183**, 1665-1681.e18.

Liu,Y. *et al.* (2023) Patient-derived xenograft models in cancer therapy: technologies and applications. *Signal Transduction and Targeted Therapy 2023 8:1*, **8**, 1–24.

Macosko,E.Z. *et al.* (2015) Highly parallel genome-wide expression profiling of individual cells using nanoliter droplets. *Cell*, **161**, 1202–1214.

Mölder,F. *et al.* (2021) Sustainable data analysis with Snakemake. *F1000Research 2021 10:33*, **10**, 33.

Rodriques,S.G. *et al.* (2019) Slide-seq: A scalable technology for measuring genome-wide expression at high spatial resolution. *Science (1979)*, **363**, 1463–1467.

Smith,T. *et al.* (2017) UMI-tools: modeling sequencing errors in Unique Molecular Identifiers to improve quantification accuracy. *Genome Res*, **27**, 491–499.

Ståhl,P.L. *et al.* (2016) Visualization and analysis of gene expression in tissue sections by spatial transcriptomics. *Science (1979)*, **353**, 78–82.

Stickels,R.R. *et al.* (2021) Highly sensitive spatial transcriptomics at near-cellular resolution with Slide-seqV2. *Nat Biotechnol*, **39**, 313–319.

Sztanka-Toth,T.R. *et al.* (2022) Spacemake: processing and analysis of large-scale spatial transcriptomics data. *Gigascience*, **11**, 1–14.

The HDF Group The HDF5® Library & File Format - The HDF Group.

Vo,T. *et al.* (2023) Spatial transcriptomic analysis of Sonic hedgehog medulloblastoma identifies that the loss of heterogeneity and promotion of differentiation underlies the response to CDK4/6 inhibition. *Genome Med*, **15**, 1–28.

Wolf,F.A. *et al.* (2018) SCANPY: Large-scale single-cell gene expression data analysis. *Genome Biol*, **19**, 1–5.

Woo,X.Y. *et al.* (2019) Genomic data analysis workflows for tumors from patient-derived xenografts (PDXs): Challenges and guidelines. *BMC Med Genomics*, **12**, 1–19.

Wu,S.Z. *et al.* (2021) A single-cell and spatially resolved atlas of human breast cancers. *Nat Genet*, **53**, 1334–1347.

Zentgraf,J. and Rahmann,S. (2021) Fast lightweight accurate xenograft sorting. *Algorithms for Molecular Biology*, **16**, 1–16.

Zhu,Q. *et al.* (2018) Identification of spatially associated subpopulations by combining scRNAseq and sequential fluorescence in situ hybridization data. *Nature Biotechnology 2018 36:12*, **36**, 1183–1190.

**List of Supplementary Figures:**

**Supplementary Fig 1: Overview of the Xenomake Pipeline.** The graph shows all the processing, mapping, xenograft sorting, expression, and downstream processing performed by Xenomake. Arrows show the order of rules performed starting with a FASTQ file of PDX SRT experiment (**Input**). The **outputs** are a spot-by-gene experiment matrix for graft genome, and a spot-by-gene expression matrix for host genome. For our case, the graft will be human, and the host will be mouse, since this is the most common PDX set up.

**Supplementary Fig 2**: **Results of applying Xenomake on Medulloblastoma PDXs**. **a**. Palbociclib treated, **b**. control setting. The spatial distribution of total mouse (stroma) and human (epithelium) mRNA reads is visualized.

**Supplementary Fig 3: Comparison between Xenomake and Space Ranger on Medulloblastoma PDXs.** **a**. Medulloblastoma PDX (control) correlation between Xenomake and Space Ranger in total UMI count per gene. **b**. Medulloblastoma PDX (palbociclib-treated) dataset, correlation between Xenomake and Space Ranger in total UMI count per gene. **c-d**. Number of genes detected per spatial barcode for various detection thresholds (UMI count>=1, 2, 3, 4, 5). UMI.ge.1 stands for UMI greater than or equal to 1. **c.** Medulloblastoma PDX (palbo) dataset. **d**. Medulloblastoma PDX (control) dataset.

**Supplementary Fig 4**: **Analysis of increased aligned reads in Xenomake in TNBC dataset**. Venn diagrams depicting the comparison between Xenomake and Space Ranger aligned reads in human (left) and mouse (right) portions. Xenomake improvement, corresponding to the Xenomake-specific fraction in the Venn Diagram, is found to be derived from up to 80% unambiguously assigned reads (from Xengsort). Ambiguous/both category makes up the remaining 15-20%.

**Supplementary Fig 5: Comparison between Xenomake and Space Ranger on specific genes in TNBC and scRNAseq validation. a.** Scatter plot showing the total gene expression (across barcodes) in Xenomake vs. Space Ranger generated mouse gene expression matrices. Each dot: a mouse gene. Discrepant genes (labeled) are largely grouped into Space Ranger-high genes (see blue fonts), and Xenomake-high genes (see blue fonts). **b**. Workflow of scRNAseq validation. For each discrepant gene-set, we looked in the Wu et al 2021 breast cancer scRNAseq atlas which provides cell-type specific expression for any genes of interest. We are interested in whether the discrepant genes have evidence of stroma cell-type specific expression in scRNAseq data, because these genes are mouse genes representing the stroma. **c**. Results of validation on Xenomake-high mouse genes. Red boxes show genes with evidence of stroma cell-type expression according to scRNAseq. **d**. Results of validation on Space Ranger-high mouse genes. Blue boxes: genes showing non-specificity in epithelial cancer cells. **e**. Stroma cell-type specificity scores, measured in entropy. **Lower means better**. Red dot and vertical bar: mean +/- 1 standard deviation. **f**. Entropy divided by cell-types. Lower means better. Dot: mean.

**Supplementary Fig 6**: **Xenomake identifies stroma (mouse) vs. epithelium (human) biased gene expression among cell-type marker genes and cytokines.** Expression of a homologous gene pair is compared (for example: Csf1r vs CSF1R, and it is labeled as Csf1r in the plot). a. Cell-type marker genes. Labeled genes indicate examples of those showing compartment biased expression. b. Cytokine genes.
