# Supplementary material for: Xenomake: a pipeline for processing and sorting xenograft reads from spatial transcriptomic experiments
Source: Bioinformatics. 2024 Oct 14;40(11):btae608. doi: 10.1093/bioinformatics/btae608 (PMC11583937; doi:10.1093/bioinformatics/btae608)

Supplementary Fig 1

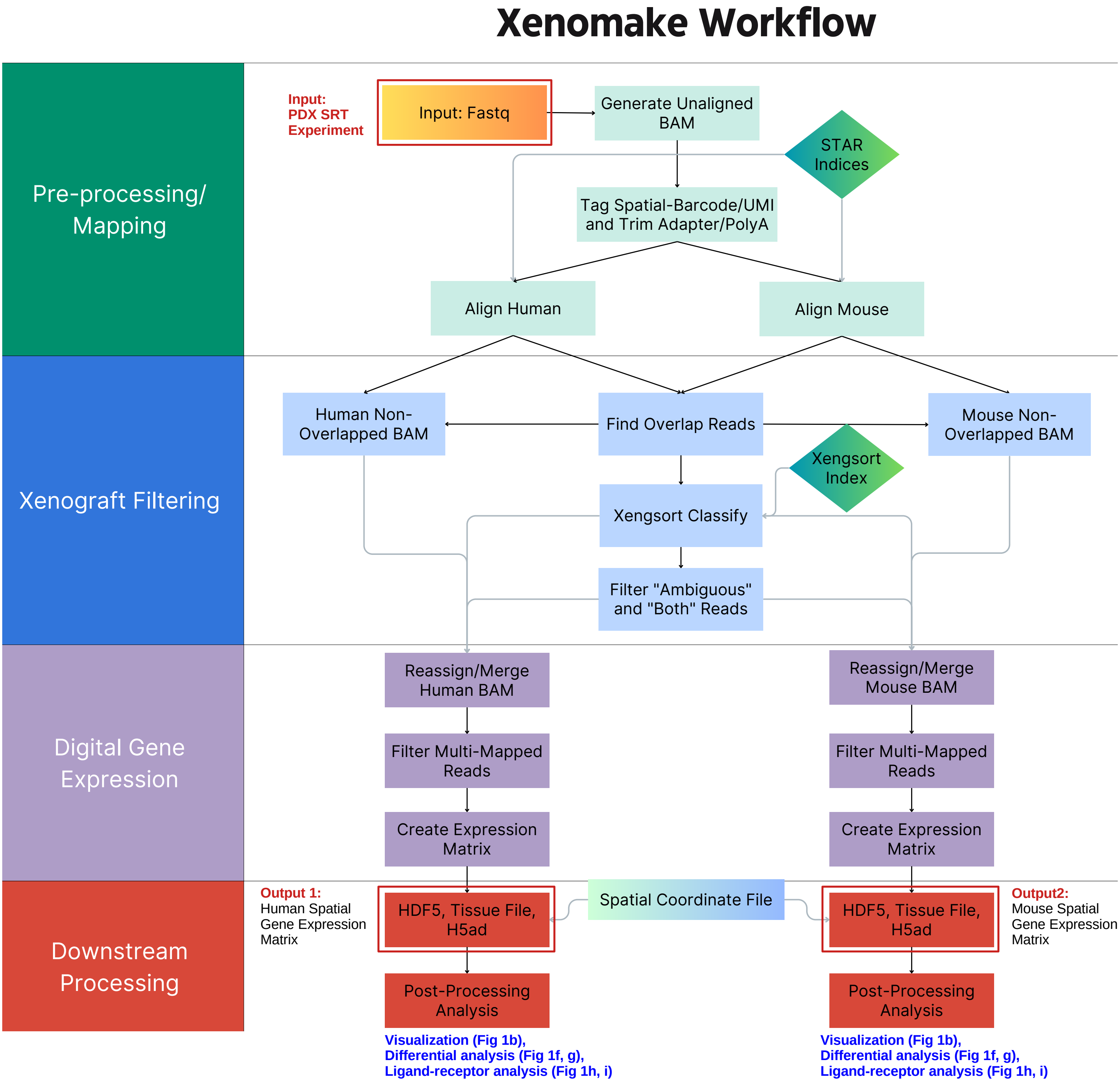

Supplementary Fig 2

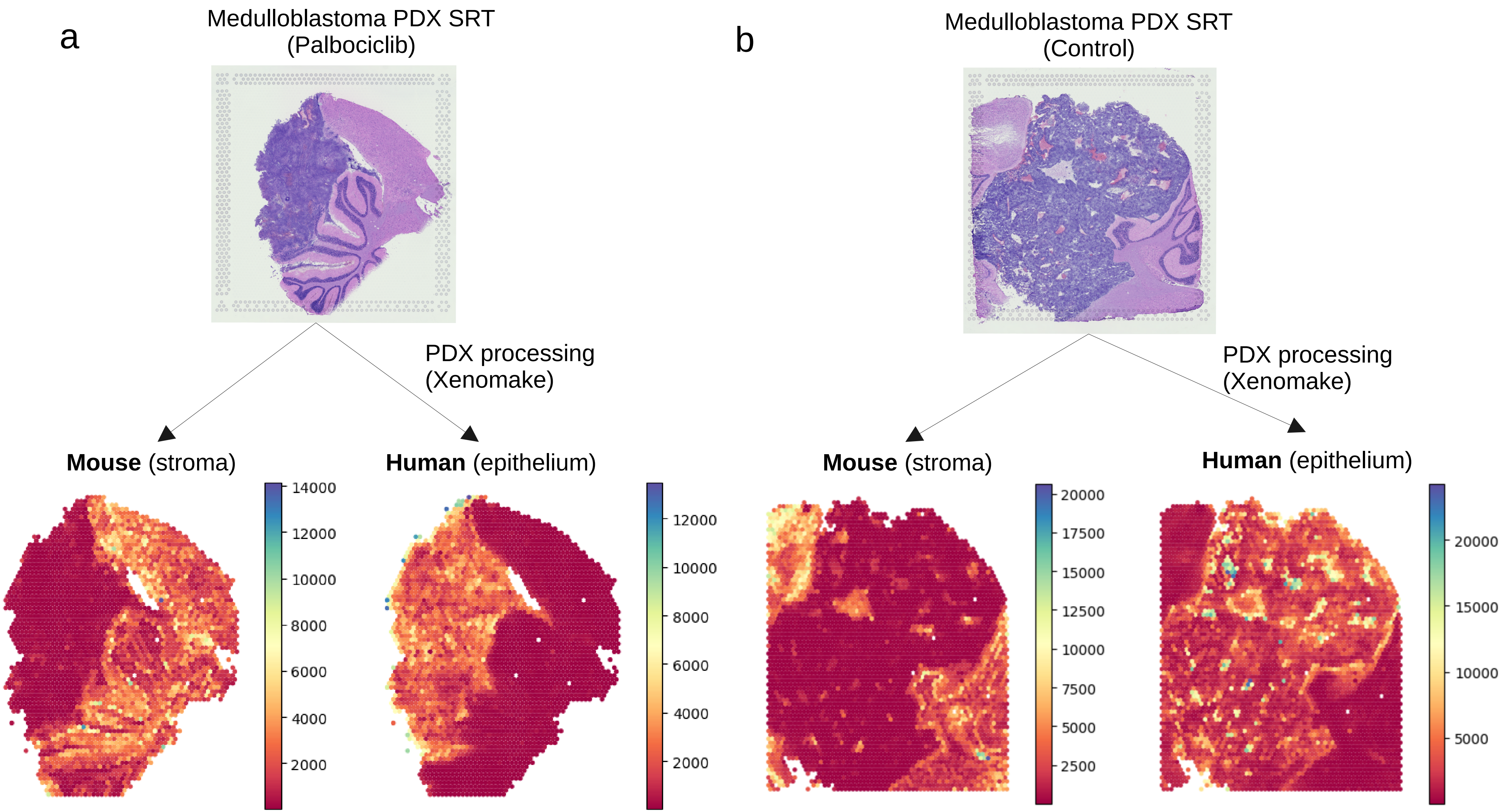

Supplementary Fig 3

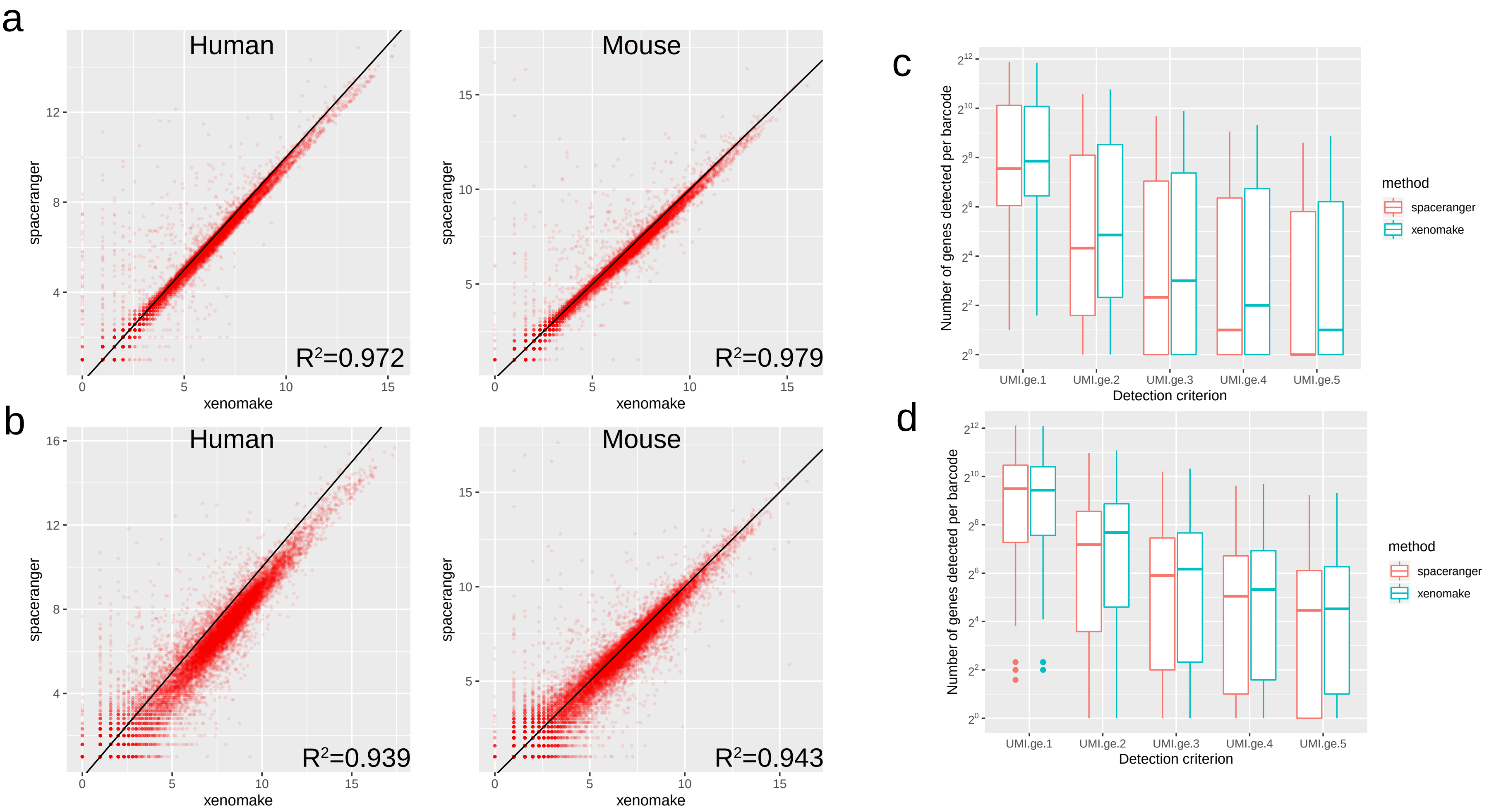

Supplementary Fig 4

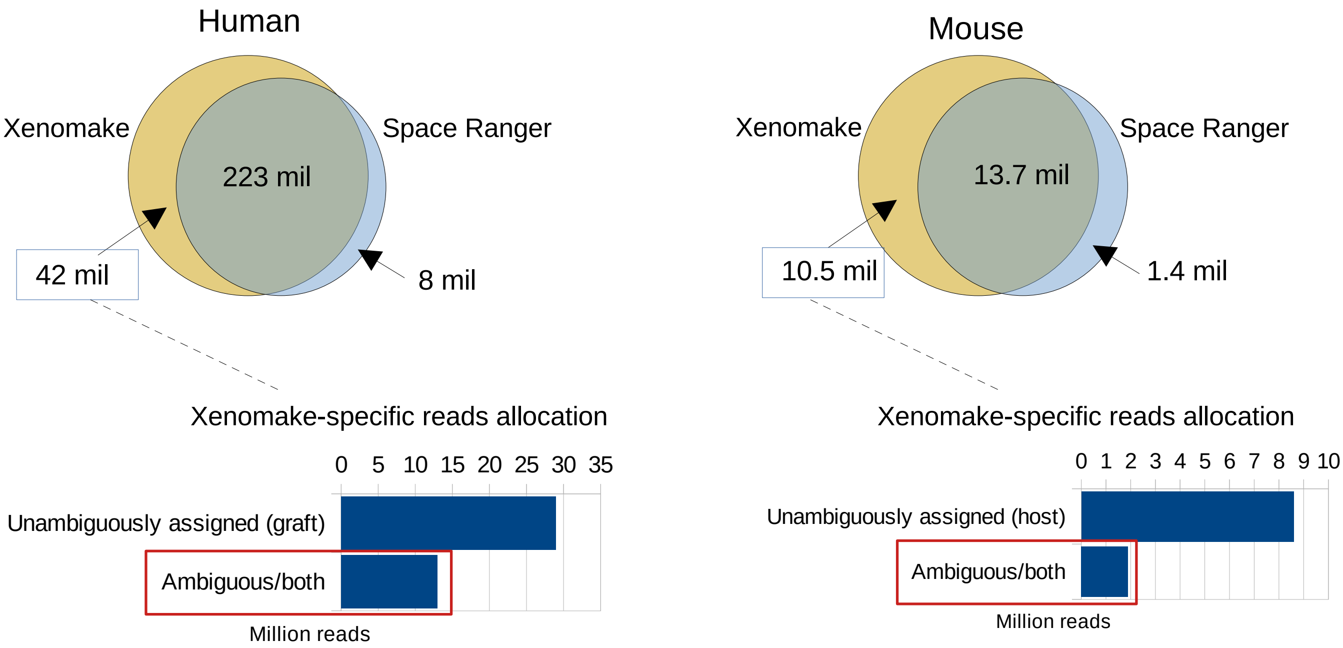

Supplementary Fig 5

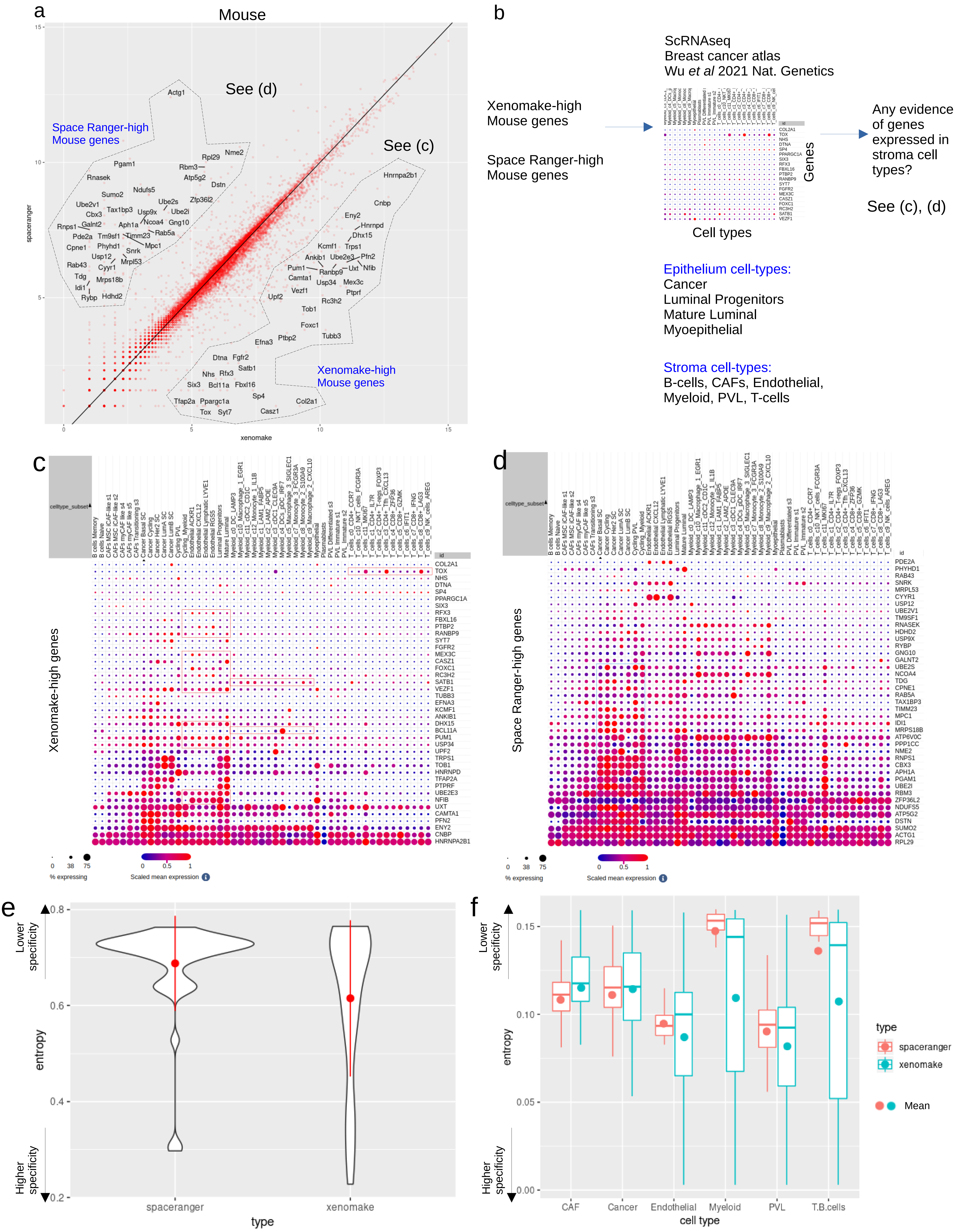

Supplementary Fig 6

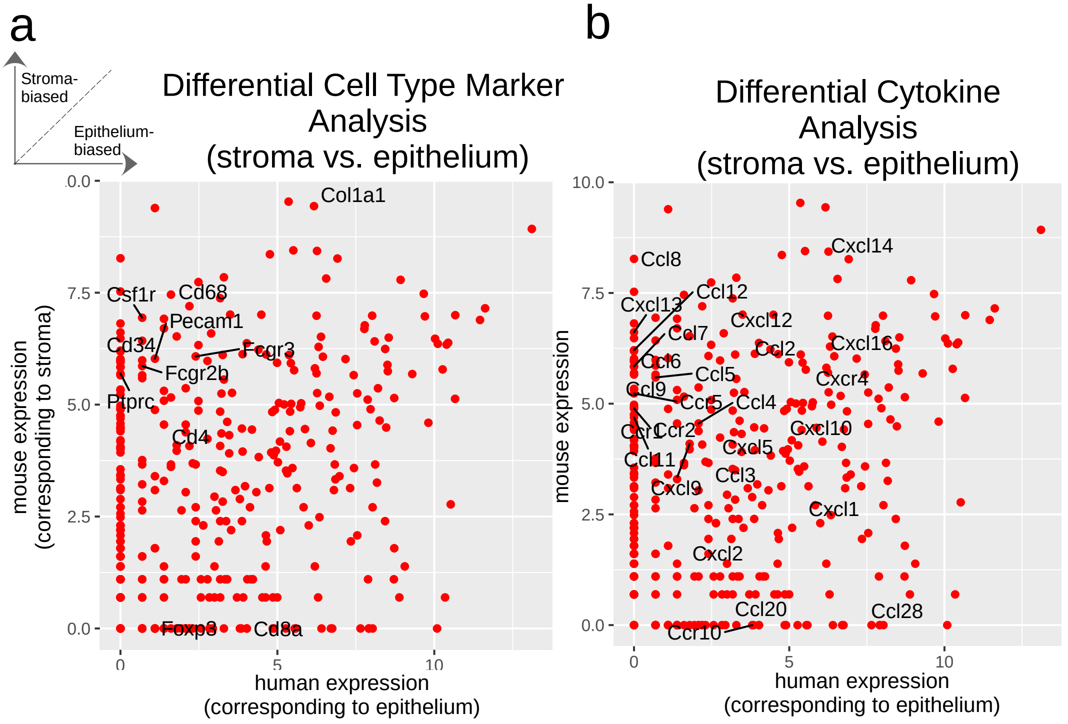

Supplement: btae608_Supplementary_Data [file btae608_supplementary_data.zip › combinepdf.supp (1).pdf]
